# Genetic engineering of Clostridium thermocellum DSM1313 for enhanced ethanol production

**DOI:** 10.1186/s12896-016-0260-2

**Published:** 2016-05-11

**Authors:** Saranyah Kannuchamy, Nisha Mukund, Lilly M. Saleena

**Affiliations:** Department of Bioinformatics, School of Bioengineering, SRM University, Kattankulathur, 603203 Tamil Nadu India

**Keywords:** *Clostridium thermocellum*, homoethanol pathway, *Zymomonas mobilis*, *pdc*, *adh*

## Abstract

**Background:**

The twin problem of shortage in fossil fuel and increase in environmental pollution can be partly addressed by blending of ethanol with transport fuel. Increasing the ethanol production for this purpose without affecting the food security of the countries would require the use of cellulosic plant materials as substrate. *Clostridium thermocellum* is an anaerobic thermophilic bacterium with cellulolytic property and the ability to produce ethanol. But its application as biocatalyst for ethanol production is limited because pyruvate ferredoxin oxidoreductase, which diverts pyruvate to ethanol production pathway, has low affinity to the substrate. Therefore, the present study was undertaken to genetically modify *C. thermocellum* for enhancing its ethanol production capacity by transferring pyruvate carboxylase (*pdc*) and alcohol dehydrogenase (*adh*) genes of the homoethanol pathway from *Zymomonas mobilis.*

**Results:**

The *pdc* and *adh* genes from *Z. mobilis* were cloned in pNW33N, and transformed to *Clostridium thermocellum* DSM 1313 by electroporation to generate recombinant CTH-*pdc,* CTH-*adh* and CTH-*pdc-adh* strains that carried heterologous *pdc*, *adh*, and both genes, respectively. The plasmids were stably maintained in the recombinant strains. Though both *pdc* and *adh* were functional in *C. thermocellum*, the presence of *adh* severely limited the growth of the recombinant strains, irrespective of the presence or absence of the *pdc* gene. The recombinant CTH-*pdc* strain showed two-fold increase in pyruvate carboxylase activity and ethanol production when compared with the wild type strain.

**Conclusions:**

Pyruvate decarboxylase gene of the homoethanol pathway from Z *mobilis* was functional in recombinant *C. thermocellum* strain and enhanced its ability to produced ethanol. Strain improvement and bioprocess optimizations may further increase the ethanol production from this recombinant strain.

## Background

Blending of ethanol with fossil fuel is recommended to address the problem of increasing demand for transportation fuel without environmental pollution. This implementation was planned because ethanol is derived from renewable biological sources, and has promising properties such as anti-knock potential [1, 2] and cleaner combustion [3, 4]. Several countries around the world are adopting this strategy, and are planning to increase the percentage of ethanol blending in a phased manner [5]. In this context, allocation of natural resources for ethanol production in place of food production, and completing interests in ethanol for industrial and potable purposes are the major concerns to be addressed. For example, sugarcane molasses serves as the sole feedstock for bioethanol production in India where the annual production is approximately 2.7 billion litres of which only 30 % is offered for fuel purposes [6, 7]. When considering the supply of feedstock, increasing the production of ethanol from sugarcane molasses is questionable because land allocation for sugarcane cultivation is limited [8], and it requires 20 megalitres of water per hectare [9]. Diverting land and water resources from production of food crops such as wheat and rice to sugarcane will threaten the food security of the countries. Therefore, plant materials from forests, urban wastes, and agricultural wastes were considered for their utility as feedstock for ethanol production [10].

A revolution of using cellulosic agricultural wastes such as corn stalk, wheat and rice straw, husk, and bran as feedstock for ethanol production was explored in many countries. Microorganisms having the capacity of utilizing cellulosic substrates for ethanol production were exploited for this purpose [11]. *Clostridium thermocellum* is one of the most efficient cellulolytic microorganisms due to the presence of cellulosomes that bind and metabolize cellulose and hemicellulose [12]. It is an anaerobic, thermophilic, and ethanol producing organism, which can be used as a biocatalyst for ethanol production from cellulosic substrates. However, the ethanol production pathway of *C. thermocellum* is not efficient because pyruvate ferrodoxin oxidoreductase, the key enzyme of this pathway [13], has lower affinity than lactate dehydrogenase and phosphotransacetylase towards the common substrate, pyruvate. As a result, the yield of ethanol is reduced, and other undesirable by-products like acetic acid and lactic acid accumulate [14–16].

*Zymomonas mobilis has* a distinctive Entner-Doudoroff pathway to metabolize glucose to pyruvate [14], and also a homoethanol pathway to metabolize pyruvate to ethanol. Two key enzymes of homoethanol pathway are pyruvate decarboxylase and alcohol dehydrogenase, which convert pyruvate to acetaldehyde and acetaldehyde to ethanol, respectively. Genes coding for these two enzymes from *Z. mobilis* were successfully used for metabolic engineering of enhanced ethanol production in *Geobacillus thermoglucosidasus* [15]*, Escherichia coli* [16]*, Clostridium cellulolyticum* [17]*,* and *Synechococcus sp.* [18]*.* The present study reports genetic modification of *C. thermocellum* for enhanced ethanol production by introducing the pyruvate decarboxylase and alcohol dehydrogenase genes from *Z. mobilis.* The recombinant *C. thermocellum* strains were analysed for the expression of the cloned genes and ethanol production efficiency.

## Results and discussion

The present study explored the possibility of enhancing the ethanol production capacity of *Clostridium thermocellum* by transferring pyruvate decarboxylase (*pdc*) and alcohol dehydrogenase (*adh*) genes from *Zymomonas mobilis*. The *pdc* and *adh* genes were successfully PCR amplified and cloned in pNW33N to obtain pNW33N-*pdc,* pNW33N-*adh,* and pNW33N-*pdc-adh,* which contained *pdc*, *adh* and both genes, respectively. The vector contained chloramphenicol acetyltransferase gene, which functions in gram negative and gram positive bacteria [19]. C. *thermocellum* is a difficult species for plasmid transformation due to endospore formation and strict anaerobic growth [20]. Electroporation method of plasmid transformation in *C. thermocellum* was illustrated by Olson and Lynd [21], and increasing the pulse duration and amplitude improved the transformation efficiency of larger plasmids [22]. In the present study, pNW33N-*adh* (5.3 Kb) was the smallest plasmid, followed by pNW33N-*pdc* (5.9 Kb) and pNW33N-*pdc-adh* (7.0 Kb). We have varied the pulse duration from 1.0 to 3.0 milliseconds (ms), and amplitude from 1500 to 2000 V in order to get successful transformation. A transformation efficiency of 1.5 × 10^3^/μg, 1.0 × 10^3^/μg, and 0.85 × 10^3^/μg was obtained for pNW33N-*adh*, pNW33N-*pdc,* and pNW33N-*pdc-adh* by applying 1.5 ms with 1500 V, 2.0 ms with 1600 V, and 3.0 ms with 1800 V, respectively. Bigger plasmids required longer pulse duration and higher amplitude of voltage for successful transformation. The recombinant stains were continuously sub-cultured ten times in CTFUD medium supplemented with 6.0 mg/L thiamphenicol, and genetic stability was monitored after every sub-culture (72 h each). The plasmids were found to be stably retained in the recombinant strains as determined by colony PCR amplification of plasmid-borne genes.

**Fig. 1:**
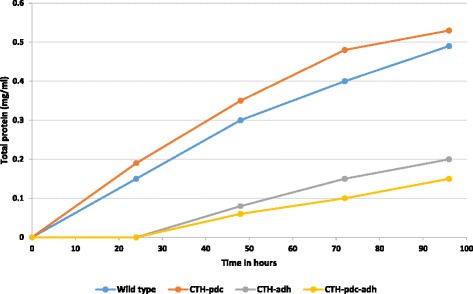
Growth pattern of wild type and recombinant CTH-*pdc,* CTH-*adh* and CTH-*pdc-adh* strains measured in terms of increase in protein content with time

Growth profile of the wild type and recombinant strains was monitored every 24 h for 96 h after inoculation. Growth of *C. thermocellum* can be visually observed by the development of a deep yellow tinge in the growth medium due to the release of an insoluble yellow affinity substance as a result of cellulose utilization by the organism [23, 24]. Wild type and the recombinant CTH-*pdc* strain developed the deep yellow tinge 24 h after inoculation indicating cellulose utilization and growth. In contrast, the recombinant CTH-*adh* and CTH-*pdc-adh* strains developed the deep yellow tinge only after 48 h. Considering the turbidity caused by the cellulose in the medium as hindrance, growth of *C. thermocellum* in CTFUD medium was monitored by estimating protein content instead of measuring the optical density. The comparative growth pattern of the wild type and recombinant strains is illustrated in Fig. 1. Growth rate of recombinant CTH-*pdc* strain was comparable with that of the wild type strain. However, the growth rate of the recombinant strain that carried either *adh* gene alone or *adh* and *pdc* genes showed significantly lower growth rate when compared with wild type or recombinant CTH-*pdc* strain. The same was observed when these constructs were present in *E. coli*, which may be due to the reverse catalysis of ethanol to acetaldehyde by alcohol dehydrogenase [25].

**Fig. 2:**
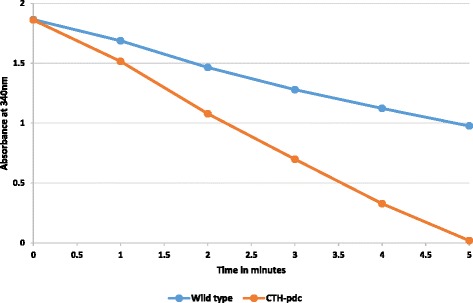
Pyruvate dependent reduction of NAD^+^ assay in wild type and recombinant CTH-*pdc* strains

Metabolically engineered *Klebsiella oxytoca* with the pyruvate decarboxylase gene from *Z. mobilis* was reported to augment its pyruvate decarboxylation capacity [26]. *C. thermocellum* contains native pyruvate ferredoxin oxidoreductase enzyme for the decarboxylation of pyruvate to which the recombinant pyruvate decarboxylase enzyme from *Z. mobilis* was added by genetic engineering. Cell free extracts from the fermented cultures of wild type and recombinant CTH-*pdc* strains were assayed for pyruvate decarboxylation based on pyruvate-dependant reduction of NAD^+^. The recombinant CTH-*pdc* strain showed faster reduction of NAD^+^ than the wild type strain (Fig. 2). Specific activity of PDC was 0.6 U/mg in the wild type and 1.8 U/mg in the recombinant CTH-*pdc*. These results showed that the pyruvate decarboxylase from *Z. mobilis* was functional in the recombinant CTH-*pdc* strain and enhanced its capacity for pyruvate decarboxylation.

Time course experiment on ethanol production by dichromate oxidation assay, and estimation of ethanol content by gas chromatography were carried out only for the recombinant CTH-*pdc* strain. Ethanol concentration remained undetectable in both wild type and CTH-*pdc* recombinant strain until 24 h. Time dependent increase in ethanol production was observed in 48 h and 72 h. There was no significant increase in ethanol production beyond 72 h of fermentation. Therefore, wild type and recombinant CTH-*pdc* strains were batch fermented for 72 h, and ethanol was solvent extracted, and analysed in gas chromatography. The CHE-*pdc* recombinant strain yielded 3.0 g/L ethanol (53 % theoretical ethanol yield) compared to 1.5 g/L ethanol (26 % theoretical ethanol yield) from the wild type strain. Earlier studies in *C. thermocellum* have shown theoretical ethanol yield of 51 % by deleting the genes for acetate dehydrogenase and phosphotransacetylase [27], and 69 % by deleting all the genes for hydrogenase activity, except *ech* gene [28]. Metabolic engineering of *Geobacillus thermoglucosidasius* with the *pdc gene* of *Z. mobilis* was also reported to increase the ethanol yield [15]. Two-fold increase in ethanol production reported in the current study due to the recombinant expression of *Z. mobilis* pyruvate decarboxylase gene in *C. thermocellum* indicates the potential of this approach towards developing *C. thermocellum* as an efficient biocatalyst for ethanol production from cellulosic plant materials. Feedback inhibition of ethanol production and accumulation of growth limiting compounds might be limiting ethanol production beyond 72 h as indicated by the declining growth rate and static ethanol production. Though there is still scope for further strain improvement by genetic engineering, bioprocess interventions such as simultaneous distillation of ethanol from the fermenting media, fed-batch method of fermentation, and optimization of media and growth conditions may further improve the productivity.

## Conclusions

Cellulolytic potential of *C. thermocellum* will be highly useful in the green chemistry approach towards the production of ethanol from cellulosic plant materials; however, its ability to produce ethanol from pyruvate is limited. The present study showed that ethanol production from *C. thermocellum* can be enhanced by transferring *pdc* gene that codes for pyruvate decarboxylase, a key enzyme in the homoethanol pathway of *Z. mobilis.* Further improvement in ethanol production can be warranted by the strain improvement and bioprocess optimization.

## Methods

### Bacterial strains

*Clostridium thermocellum* DSM1313 was obtained from DSMZ, Germany (https://www.dsmz.de). The organism was cultured and maintained with CTFUD broth and agar media, which comprised of the following components in g/L; sodium citrate 3.0, ammonium sulphate 1.3, calcium chloride 0.13, L-cysteine 0.5, β-glycerolphosphate 6.0, ferrous sulphate 0.001, cellobiose 10.0, yeast extract 4.5, magnesium chloride 2.6 and potassium dihydrogen phosphate 1.5. The pH of the medium was maintained at 7.0. Stoppered vials and bottles were used for culturing and maintaining the organism. Cellulose or cellobiose was used as the carbon source. Nitrogen gas was used to maintain anaerobic condition. The incubation temperature was maintained at 60 °C in a static condition. *Zymomonas mobilis* was obtained from ATCC, USA (http://www.atcc.org). The organism was inoculated in RM medium (pH 6.0), which comprised the following components in g/L; yeast extract 10.0, potassium dihydrogen phosphate 2.0 and glucose 20.0. It was grown in the culture flasks kept in orbital shaker at room temperature and 250 rpm.

### Vector construction

DNA isolation, PCR amplification and cloning were done by following standard molecular cloning protocols [29]. Gene-specific primers for pyruvate decarboxylase (GenBank Acc. No. HM235920) with 5′ *Bam*HI site and 3′*Xma*I site, and alcohol dehydrogenase (GenBank Acc. No. M15394) with 5′ *Kpn*I and 3′ *Eco*RI site were synthesised, and the respective genes were amplified by PCR using the genomic DNA of *Z. mobilis* as template*.* The PCR amplified genes were restriction digested and cloned in pNW33N [19], which contained cellobiose phosphorylase promoter [30] and ribosome binding site to facilitate translation [31]. Three separate constructs, each with pyruvate decarboxylase gene (pNW33N-*pdc) or* alcohol dehydrogenase gene (pNW33N-*adh)* or both genes (pNW33N-*pdc-adh*) were transformed to *E. coli* BL21, and maintained under chloramphenicol selection (34.0 mg/L).

### Transformation of *C. thermocellum*

The plasmid DNA of pNW33N-*pdc*, pNW33N-*adh* and pNW33N-*pdc-adh* were isolated from *E. coli* BL21, and transformed to *C. thermocellum* by electrotransformation using GenePulser Xcell Electroporator (Bio-Rad, USA). Cells of *C. thermocellum* were grown to an optical density of 0.8 at 600 nm, and 1.0 ml of the culture was transferred to centrifuge tube, and chilled on ice for 10 min. The culture was centrifuged at 6500 rpm for 5 min, and the pellet was washed twice with a wash buffer containing 250 mM sucrose and 10 % glycerol, and resuspended in 300 μl of the wash buffer. All the above mentioned steps were carried out in an anaerobic cabinet. The cell suspension (30 μl) and plasmid DNA (100 ng) were taken in an electroporation cuvette, and desired square pulse and amplitude were applied. The electroporated cells were immediately retrieved, and transferred to CTFUD medium supplemented with 1X concentration of vitamin solution (1000X; pyridoxamine hydrochloride 2000 mg/L, biotin 200 mg/L, and aminobenzoic acid 400 mg/L, and vitamin B12 200 mg/L). The cells were incubated at 51 °C for 16 h, and plated in CTFUD agar medium supplemented with 6.0 mg/L thiamphenicol. The transformed colonies were screened by colony PCR using gene speciifc primers, and confirmed by plasmid isolation and restriction digestion. Recombinant *C. thermocellum* containing pNW33N-*pdc*, pNW33N-*adh* and pNW33N-*pdc-adh* plasmids were named as CTH-*pdc,* CTH-*adh* and CTH-*pdc-adh*, respectively. Recombinant *C. thermocellum* strains were always maintained in the presence of thiamphenicol (6.0 mg/L).

### Enzyme assay

The wild and recombinant CTH-*pdc* were grown to an optical density of 0.8 at 600 nm, and 10 ml of the culture was transferred to centrifuge tubes. The culture was centrifuged at 6,000 rpm for 10 min, and the pellet was resuspended in 0.5 ml assay buffer (0.1 M Tris–HCl, 0.1 mM FeSO4 and 0.1 mM DTT, pH 7.5). The cells were lysed using 20 μl of lysozyme (10 mg/ml), and the resulting lysate was clarified by treating it with 5.0 units of DNase I. The lysate was centrifuged at 12,000 rpm for 10 min at room temperature, and the cell free extract (supernatant) was used for enzyme assays. Pyruvate decarboxylase was assayed by measuring pyruvate-dependant reduction of NAD^+^ in the presence of alcohol dehydrogenase as the coupling enzyme [32]. Protein concentration was estimated by Bradford method [33]. One unit of enzyme represents the amount of enzyme required for conversion of 1.0 μmol of substrate per minute into specific products. Specific activity of the enzyme was calculated by using the extinction coefficient of NAD^+^ (6.22 cm^−1^ mM^−1^) [34].

### Fermentation and analysis of ethanol production

Fresh inoculum was prepared by adding 1 % of stock inoculum to 500 ml of CTFUD medium, and fermenting the culture at 52 °C for 96 h without stirring under Nitrogen sparging. The wild type and recombinant CTH-*pdc,* CTH-*adh* and CTH-*pdc-adh* strains were inoculated in the ratio of 1:10 with fresh CTFUD medium in replicates. Batch fermentation was done in 48 stoppered vials, and analysed for ethanol production by dichromate oxidation method [35] at 24, 48, 72 and 96 h after inoculation. The cultures were immediately transferred to 4 °C and maintained at the same temperature to avoid the loss of ethanol during storage. Ethanol present in the fermented culture was extracted in tri-n-butyl phosphate and estimated by potassium dichromate oxidation method [36]. Ethanol concentration in the solvent was also measured by gas chromatographic analysis by using flame ionization detector with column at 200 °C. Nitrogen was used as the carrier gas (30 ml/min), and *n*-butanol was used as internal standard for the quantitative analysis.
